# Distribution of atherosclerotic stenosis determining early neurologic deterioration in acute ischemic stroke

**DOI:** 10.1371/journal.pone.0185314

**Published:** 2017-09-25

**Authors:** Seung-Jae Lee, Dong-Geun Lee

**Affiliations:** 1 Department of Neurology, Soonchunhyang University Bucheon Hospital, Bucheon, South Korea; 2 Department of Neurology, Sejong General Hospital, Bucheon, South Korea; Universitatsklinikum Freiburg, GERMANY

## Abstract

**Background and purpose:**

Early neurologic deterioration (END) during the acute stage of stroke is clinically important because of its association with poor outcomes. The purpose of this study was (1) to investigate variables associated with END, (2) to determine the distribution of atherosclerotic stenosis associated with END, and (3) to clarify the relationship between END and clinical outcomes.

**Methods:**

516 patients with acute ischemic stroke were included. The median follow-up period was 31.7 months. END was defined as a ≥2 point increase in the National Institutes of Health Stroke Scale (NIHSS), ≥1 point increase in level of consciousness or motor item of the NIHSS, or the development of any new neurological deficits during the first 72 hours of hospitalization. A signal loss on 1.5-T magnetic resonance angiography exceeding 50% was considered to be significant for the categorization of stenosis pattern.

**Results:**

The prevalence of END was 19.0%. END was associated with intracranial atherosclerotic stenosis (IAS) together with large artery atherosclerosis (LAA) subtype. In particular, stenosis of basilar artery or posterior cerebral artery was independently associated with END. Lesion growth or hypoperfusion was more accountable for END in patients with IAS, whereas intracerebral hemorrhage or edema/herniation was more frequently observed in END patients without IAS. Patients with END had a higher rate of mortality, but a similar rate of further vascular events compared to patients without END.

**Conclusion:**

Pre-stroke IAS and LAA subtype could determine the development of END during the acute stage of ischemic stroke.

## Introduction

Neurologic worsening during the early period of stroke, which is referred to as early neurologic deterioration (END), is clinically crucial because it is frequently encountered in real-world stroke practice and is associated with poor clinical outcomes [1, 2]. Many studies have investigated the factors associated with END. Reported factors include advanced age [3], initial stroke severity [1–3], high blood glucose values or history of diabetes mellitus [1, 3, 4], blood pressure variability [5], occlusion of internal carotid (ICA) or middle cerebral artery (MCA) [6, 7], symptomatic steno-occlusive arterial disease [8], and ICA territory infarct [9]. However, only one study has examined the distribution of atherosclerotic stenosis (intracranial versus extracranial); the study included only symptomatic stenotic lesion [8].

Intracranial atherosclerotic stenosis (IAS) is important in stroke management, especially in Asian countries where IAS comprises 30–50% of strokes [10]. Patients with IAS have higher recurrence rates of stroke and death than those without [11]. Considering its anatomical location, intracranial collateral channels may be more limited with IAS compared to extracranial atherosclerotic stenosis (EAS), leading to stagnated flow in relevant arterial territories and thus resulting in decreased washout of emboli [12, 13].

Accordingly, we hypothesized that IAS interrupts early recovery of perfusion into the peri-infarct area during the acute period of stroke, which causes END. This study explored the hypothesis by investigating clinical variables associated with END, determining if the distribution of atherosclerotic stenosis is associated with END, and clarifying the relationship between END and long-term clinical outcomes (functional outcome, long-term survival, and further vascular events).

## Methods

### Ethics statement

The Institutional Review Board of Sejong General Hospital approved this study with informed consent waived.

### Patients

A prospective stroke registry database was used for the retrospective investigation of 661 acute ischemic stroke patients who were consecutively admitted to Sejong General Hospital within 7 days after symptom onset between January 2011 and September 2016. Patients were excluded if they presented > 48 hours after being last seen as normal (n = 124) or had no data of brain vessel or 6-month outcome (n = 21). Finally, 516 patients were included for the analysis.

All survivors were principally followed-up by outpatient clinic attendance. However, 104 patients were not followed-up by our clinic at the time of this study. Of these, the conditions of 81 patients were ascertained by telephone contact with the patients or their relatives. The other 23 patients were censored at the last clinic visit. Modified Rankin scale (mRS) score at 6 months was determined using a structured interview for accurate grading according to the Korean Clinical Research Center for Stroke [14].

We monitored mortality and major vascular events (stroke, acute coronary syndrome, or peripheral artery occlusion) after index stroke. The nature of the vascular event was principally based on medical records from the treating physician at Sejong General Hospital. In the absence of records, medical information was acquired from treating physicians at other institutions. Uncertain information was excluded from the study.

### Definitions

Ischemic stroke was defined as a focal neurologic deficit of an abrupt onset lasting > 24 hours with evidence of new infarct lesions on brain imaging. All the strokes were classified as large artery atherosclerosis (LAA), cardioembolism, lacune, two or more mechanisms, cryptogenic stroke, and other causes according to the Trial of Org 10172 in Acute Stroke Treatment subtype classification system [15]. Branch atheromatous disease (BAD) was defined as an elongated infarct lesion extending from the origin of penetrating artery territory with any discernible stenosis of relevant parent artery (mainly M1 or basilar artery), and was considered as LAA [16]. Metabolic syndrome was defined as the presence of any 3 of 5 risk factors: elevated waist circumference (≥90 cm in men, ≥80 cm in women), triglycerides ≥150 mg/dL, reduced high-density lipoprotein cholesterol (<40 mg/dL in men, <50 mg/dL in women), elevated blood pressure (systolic ≥130 and/or diastolic ≥85 mmHg), and fasting glucose ≥100 mg/dL, with drug treatment for each risk factor being an alternate indicator [17]. END was defined as an increase of 2 points or more in the National Institutes of Health Stroke Scale (NIHSS) score, an increase of 1 point or more on the level of consciousness or motor items of the NIHSS, or the development of any new neurological deficits during the first 72 hour of hospitalization [5, 6]. The causes of END were classified as new non-border zone lesion separate in space from a previous lesion (new lesion), lesion growth, lesion progression in border zone (hypoperfusion), intracerebral hemorrhage (ICH), brain edema or herniation, medical condition (infection, cardiac disease, etc), seizure, and undetermined.

### Risk factor assessment and brain imaging

Clinical information included age, gender, history of hypertension, diabetes mellitus and hyperlipidemia (defined as a total cholesterol level > 200 mg/dL or a low-density lipoprotein cholesterol > 130 mg/dL at the time of presentation or a history of treatment), current cigarette smoking, previous history of stroke and ischemic heart disease (defined as a known history or clinical demonstration of myocardial infarction or angina pectoris), atrial fibrillation, valvular heart disease, heavy alcohol consumption (>26 Soju drinks/month; about 20% alcohol), congestive heart failure (ejection fraction <40% on admission echocardiography with a previous episode of symptomatic heart failure characterized by dyspnea, cardiomegaly, and pulmonary edema), medication use (anthrombotics and statin) for ≥ 3 months at stroke onset, and NIHSS score at admission. All 516 patients underwent routine 12-lead electrocardiography, echocardiography, and 24-hour Holter monitoring. All patients underwent 1.5-T magnetic resonance imaging (MRI) on admission. The MRI consisted of the diffusion-weighted image, gradient echo image, fluid-attenuated inversion recovery image, three-dimensional time-of-flight intracranial MR angiography (MRA), and contrast-enhanced MRA including extracranial carotid and vertebral arteries. More than 50% signal loss on MRA was considered significant to the categorization of a stenosis pattern. Complete occlusion relevant to the infarct area was not counted as an atherosclerotic steno-occlusion if the patient had a high-risk cardioembolic source, and was thus classified as cardioembolism. Stenoses of brain vessels on MRA were classified as IAS or EAS, based on the location of the arterial stenosis, which included intracranial [intracranial ICA, MCA, anterior cerebral artery (ACA), intracranial vertebral artery (VA), basilar artery (BA), posterior cerebral artery (PCA)] and extracranial (extracranial ICA and VA).

### Data analyses

Statistical analyses were performed with SPSS software, version 18.0 (SPSS Inc., Chicago, IL). The independent t-test or Chi-square test was used to compare the difference between patients with and without END or IAS. Logistic regression analysis was performed to analyze variables associated with END and 6-month mRS ≥ 3. Kaplan-Meier survival curves were plotted for death or further major vascular events in patients with and without END. Differences in the outcomes were evaluated using the log-rank test. A Cox proportional hazards model was used to analyze variables associated with risk of mortality. Variables with *P* <0.1 in the univariate analysis were used in multivariate analysis. Unadjusted and adjusted odds ratios (ORs), hazards ratios, and 95% confidence intervals (CIs) were obtained. *P*-values < 0.05 were considered to be statistically significant.

## Results

The mean age at admission of the 516 patients (270 men and 246 women) was 67.5 years (range 18–94). Of these patients, 98 (19.0%) had an END. Table 1 presents the comparison data of patients with and without END. Patients with END were more often older, women, hypertensive, and had a higher level of blood glucose (initial and fasting), higher leukocyte count, and higher NIHSS score at admission compared to patients without END. In addition, they were more likely to have atherosclerotic stenosis and a poor functional outcome (6-month mRS ≥3). Furthermore, the association of END with poor functional outcome was significant in unadjusted and adjusted logistic regression analyses (S1 Table). There was a significant difference in the proportion of stroke subtypes between patients with and without END, with 39.8% of patients with END versus 18.4% of patients without END having LAA. In particular, BAD was closely associated with END (13 of 21 patients (61.9%) with BAD versus 85 of 495 patients (17.2%) without, *P* < 0.001).

**Table 1:** Basic characteristics of patients with and without early neurologic deterioration (END): n (%) or mean±SD.

	END (+)	END (-)	*P*
	N = 98	N = 418	
Age ≥65 years	72 (73.5)	248 (59.3)	0.009
Women	58 (59.2)	188 (45.0)	0.011
Hypertension	74 (75.5)	272 (65.1)	0.048
Diabetes	37 (37.8)	120 (28.7)	0.080
Hyperlipidemia	49 (50.0)	216 (51.7)	0.765
Current smoking	22 (22.4)	116 (27.8)	0.286
Previous stroke	18 (18.4)	68 (16.3)	0.616
Ischemic heart disease	22 (22.4)	91 (21.8)	0.884
Atrial fibrillation	31 (31.6)	153 (36.6)	0.355
Valvular heat disease	17 (17.3)	91 (21.8)	0.333
Heavy alcohol consumption	11 (11.2)	76 (18.2)	0.098
Congestive heart failure	15 (15.3)	54 (12.9)	0.532
Previous medication			
Antiplatelet	44 (44.9)	161 (38.5)	0.245
Anticoagulant	12 (12.2)	67 (16.0)	0.349
Statin	29 (29.6)	109 (26.1)	0.479
Initial systolic blood pressure (mmHg)	140.1±28.8	136.3±25.8	0.197
Initial diastolic blood pressure (mmHg)	78.5±16.9	77.4±15.4	0.529
Metabolic syndrome	53 (54.1)	190 (45.5)	0.124
Waist circumference (cm)	84.5±10.1	84.7±9.9	0.878
Triglycerides (mg/dL)	101.9±49.6	118.8±85.9	0.065
HDL-C (mg/dL)	48.2±15.5	47.7±14.5	0.751
Blood sugar, fasting (mg/dL)	130.1±40.9	116.3±40.5	0.003
Total cholesterol (mg/dL)	162.6±43.2	167.7±42.6	0.292
LDL-C (mg/dL)	102.3±37.3	106.5±38.3	0.331
Leukocyte count (x10^9^/L)	9.1±3.7	8.1±3.3	0.016
Hemoglobin (g/dL)	13.0±2.2	13.5±2.4	0.056
Platelet count (x10^9^/L)	214.3±71.4	213.7±70.0	0.698
Blood glucose at admission (mg/dL)	156.0±66.9	140.8±66.6	0.043
Initial NIHSS	11.8±10.0	5.7±7.1	<0.001
Atherosclerotic stenosis	69 (70.4)	191 (45.7)	<0.001
Stroke classification			<0.001
Large artery atherosclerosis	39 (39.8)	77 (18.4)	<0.001
Cardioembolism	29 (29.6)	170 (40.7)	0.043
Lacune	9 (9.2)	71 (17.0)	0.055
Two or more	18 (18.4)	64 (15.3)	0.456
Cryptogenic	1 (1.0)	33 (7.9)	0.014
Other causes	2 (2.0)	3 (0.7)	0.229
Reperfusion therapy (use of tPA or thrombectomy)	5 (5.1)	27 (6.5)	0.616
Poor functional outcome	73 (74.5)	105 (25.1)	<0.001

HDL-C, high-density lipoprotein cholesterol; LDL-C, low-density lipoprotein cholesterol; NIHSS, National Institutes of Health Stroke Scale; tPA, tissue plasminogen activator

*P* was calculated by Chi-square test or independent t-test. Poor functional outcome indicates scores ≥ 3 on 6-month modified Rankin scale

In univariate logistic regression, LAA (versus all the other stroke subtypes) was significantly associated with END (*P* < 0.001, OR 2.927, 95% CI 1.822–4.703). The association remained significant in adjusted analysis (Table 2). In addition, IAS and NIHSS score were significant variables associated with END in the adjusted analyses even where variable for stroke subtype was changed.

**Table 2:** Multiple logistic regression analysis for early neurologic deterioration.

	OR (95% CI)	*P*	OR (95% CI)	*P*
Age ≥ 65 years	1.205 (0.682–2.130)	0.521	1.197 (0.677–2.116)	0.537
Female	1.170 (0.684–2.000)	0.567	1.196 (0.700–2.044)	0.512
Hypertension	1.388 (0.786–2.451)	0.259	1.281 (0.726–2.262)	0.393
Heavy alcohol consumption	0.838 (0.385–1.823)	0.656	0.743 (0.341–1.620)	0.456
Blood glucose at admission (mg/dL)	1.001 (0.997–1.004)	0.673	1.001 (0.997–1.004)	0.598
Hemoglobin (g/dL)	0.988 (0.886–1.102)	0.828	0.997 (0.894–1.112)	0.957
Leukocyte count (x10^9^/L)	1.048 (0.982–1.118)	0.162	1.043 (0.977–1.114)	0.209
Initial NIHSS score	1.080 (1.051–1.111)	<0.001	1.084 (1.052–1.116)	<0.001
Intracranial atherosclerotic stenosis	1.872 (1.037–3.381)	0.037	2.137 (1.202–3.799)	0.010
Large artery atherosclerosis^†^	2.227 (1.229–4.035)	0.008		
Cardioembolism^†^			0.574 (0.302–1.092)	0.091

OR, odds ratio; CI, confidence interval; NIHSS, National Institutes of Health Stroke Scale

^†^stroke subtype versus all other subtypes

The distribution of atherosclerotic stenosis was different between patients with and without END. Patients with END were more likely to have IAS, and more often had stenotic lesions in ACA, intracranial VA, BA, PCA and extracranial ICA compared with patients without END (Table 3).

**Table 3:** Distribution of atherosclerotic stenosis according to the presence of early neurologic deterioration (END): n (%).

	END (+)	END (-)	*P*
	N = 98	N = 418	
Distribution of stenosis			<0.001
Intracranial and extracranial	23 (23.5)	55 (13.2)	
Intracranial only	41 (41.8)	102 (24.4)	
Extracranial only	5 (5.1)	34 (8.1)	
No stenosis	29 (29.6)	227 (54.3)	
EAS	28 (28.6)	89 (21.3)	0.121
Extracranial ICA	18 (18.4)	46 (11.0)	0.047
Extracranial VA	17 (17.3)	62 (14.8)	0.534
IAS	64 (65.3)	157 (37.6)	<0.001
Intracranial ICA	19 (19.4)	63 (15.1)	0.293
MCA	31 (31.6)	95 (22.7)	0.065
ACA	21 (21.4)	47 (11.2)	0.007
Intracranial VA	21 (21.4)	42 (10.0)	0.002
BA	19 (19.4)	28 (6.7)	<0.001
PCA	37 (37.8)	72 (17.2)	<0.001

EAS, extracranial atherosclerotic stenosis; IAS, intracranial atherosclerotic stenosis; ICA, internal carotid artery; VA, vertebral artery; MCA, middle cerebral artery; ACA, anterior cerebral artery; BA, basilar artery; PCA, posterior cerebral artery

*P* calculated by Chi-square test

We investigated the locations of stenosis associated with END in a multivariate logistic analysis (Table 4). Stenotic lesion in BA or PCA was independently associated with END.

**Table 4:** Multivariate logistic regression analysis to determine the location of stenosis associated with early neurologic deterioration^†^.

Location of stenosis	OR (95% CI)	*P*
Extracranial ICA	1.412 (0.709–2.812)	0.327
Intracranial ICA	0.725 (0.380–1.382)	0.328
MCA	0.894 (0.503–1.590)	0.703
ACA	1.254 (0.657–2.391)	0.493
Intracranial VA	1.546 (0.803–2.976)	0.192
BA	2.343 (1.152–4.764)	0.019
PCA	2.267 (1.327–3.872)	0.003

ICA, internal carotid artery; MCA, middle cerebral artery; ACA, anterior cerebral artery; VA, vertebral artery; BA, basilar artery; PCA, posterior cerebral artery

^†^age group (≥ 65 years), gender, hypertension, heavy alcohol consumption, blood glucose at admission, hemoglobin, leukocyte count, initial score of the National institutes of Health Stroke Scale, and stroke subtype (cardioembolism versus other subtypes) were adjusted

The most frequent cause of END was lesion growth (32.7%), followed by edema/herniation (24.5%), new lesion (12.2%), hypoperfusion (9.2%), ICH (7.1%), undetermined (6.1%), medical condition (5.1%), and seizure (3.1%). There was a significant difference of the proportion of the cause of END between patients with and without IAS (Table 5).

**Table 5:** Comparison of the causes of early neurologic deterioration between patients with and without intracranial atherosclerotic stenosis (IAS): n (%).

	Total	IAS (+)	IAS (-)	*P* = 0.028
	N = 98	N = 64	N = 34	
New lesion	12 (12.2)	7 (10.9)	5 (14.7)	
Lesion growth	32 (32.7)	23 (35.9)	9 (26.5)	
Hypoperfusion	9 (9.2)	9 (14.1)	0 (0)	
Intracerebral hemorrhage	7 (7.1)	2 (3.1)	5 (14.7)	
Edema/hernation	24 (24.5)	13 (20.3)	11 (32.4)	
Medical condition	5 (5.1)	2 (3.1)	3 (8.8)	
Seizure	3 (3.1)	2 (3.1)	1 (2.9)	
Undetermined	6 (6.1)	6 (9.4)	0 (0)	

*P* calculated by Chi-square test

Among 98 patients with END, lesion growth or hypoperfusion was more frequently observed in those with IAS than in those without IAS (50% versus 26.5%, *P* = 0.025). In contrast, ICH or edema/herniation was more frequent in patient without IAS than with IAS (47.1% versus 23.4%, *P* = 0.017). The patients without IAS were more likely than patients with IAS to have a cardioembolic stroke type (58.8% versus 14.1%, *P* < 0.001) which had a higher initial score of NIHSS compared with non-cardioembolic subtypes among the 98 patients (19.0±10.4 versus 8.8±8.2, *P* < 0.001).

**Fig 1:**
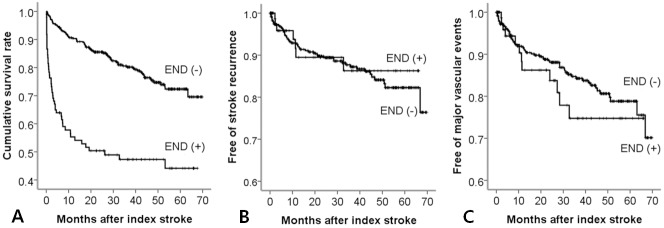
Kaplan-Meier curves for mortality, stroke recurrence and major vascular events. The patients with early neurologic deterioration had a higher rate of mortality (A: *P* < 0.001 by log-rank test), but there was no significant difference in the rate of stroke recurrence (B: *P* = 0.789 by log-rank test) and major vascular events (C: *P* = 0.411 by log-rank test) between patients with and without early neurologic deterioration.

The median follow-up period was 31.7 months (mean 31.6; range 0.1–69.8). Long-term clinical outcomes (mortality, recurrence of stroke and occurrence of major vascular events) were compared between patients with and without END (Fig 1). Patients with END had a significantly higher rate of mortality (*P* < 0.001), but there was no significant difference in the incidence of stroke recurrence and major vascular events between the two groups. When Cox proportional hazards analysis was performed to determine variables associated with mortality, END was associated with a high risk of mortality in adjusted analysis (Table 6).

**Table 6:** Cox proportional hazards models for mortality.

	Univariate		Multivariate	
	HR (95% CI)	*P*	HR (95% CI)	*P*
Age ≥65 years	2.874 (1.872–4.412)	<0.001	2.432 (1.384–4.271)	0.002
Female	1.780 (1.260–2.514)	0.001	0.863 (0.539–1.383)	0.541
Hypertension	1.097 (0.763–1.577)	0.617		
Diabetes	1.000 (0.691–1.448)	1.000		
Hyperlipidemia	0.794 (0.566–1.115)	0.183		
Previous stroke	1.271 (0.828–1.950)	0.273		
Ischemic heart disease	1.337 (0.906–1.974)	0.144		
Congestive heart failure	2.329 (1.550–3.499)	<0.001	1.251 (0.768–2.037)	0.368
Valvular heart disease	1.113 (0.741–1.671)	0.606		
Atrial fibrillation	1.506 (1.071–2.118)	0.019	0.794 (0.482–1.308)	0.365
Current smoking	0.406 (0.253–0.654)	<0.001	0.615 (0.304–1.242)	0.175
Heavy alcohol consumption	0.300 (0.152–0.590)	<0.001	0.506 (0.174–1.466)	0.209
Initial NIHSS score	1.094 (1.077–1.112)	<0.001	1.076 (1.053–1.099)	<0.001
Intracranial atherosclerotic stenosis	1.952 (1.387–2.746)	<0.001	1.005 (0.595–1.697)	0.984
Stroke subtype (cardioembolism)	1.189 (0.844–1.676)	0.321		
Early neurologic deterioration	3.599 (2.529–5.123)	<0.001	3.314 (2.041–5.383)	<0.001

HR, Hazard ratio; CI, confidence interval; NIHSS, National institutes of Health Stroke Scale

## Discussion

There are four key findings. Patients with LAA displayed a higher rate of END compared with patients with the other stroke subtypes. The intracranial distribution of atherosclerotic stenosis also had the same impact as LAA and, even without regard to the stroke symptoms, was associated with END. Of the atherosclerotic stenoses, stenotic lesion in BA or PCA was independently associated with END even after adjusting covariates. Finally, among the causes of END, lesion growth or hypoperfusion was associated with IAS.

Although LAA stroke subtype, particularly BAD, has been associated with END [4, 6, 7, 18, 19], the present data describe for the first time that the distribution of atherosclerotic stenosis (IAS) is related to END. This could be partially explained by the fact that IAS had a significant relationship to a higher rate of atherosclerotic vascular risk factors in our cohort (S2 Table) and in other studies [11, 20]. Advanced age and other risk factors may have contribution to thrombotic progression leading to END in patients with IAS. In addition, when we analyzed patients who had only IAS or EAS (i.e., excluding patients having IAS and EAS simultaneously or no stenosis), patients with IAS had more arterial stenotic lesions in brain vessels than patients with EAS only (2.7±1.8 versus 1.6±0.9, *P* < 0.001). Furthermore, brain region affected by IAS is more likely to have a limited collateral blood flow than a region affected by EAS [12, 21]. In other words, IAS may interrupt effective intracranial collateral channels (e. g. anterior or posterior communicating artery), thus causing only pial or meningeal to pial collateral available, whereas in EAS, collaterals across the circle of Willis may be preserved allowing the perfusion into a brain region distal to the occlusion.

In agreement with the above explanation, lesion growth or hypoperfusion was more accountable for END in patients with IAS than in patients without IAS. In contrast, ICH or edema/herniation was more frequently observed in END patients without IAS, who had a higher prevalence of cardioembolism associated with a higher initial stroke severity based on NIHSS score.

Among each lesion of IAS, stenotic lesion in BA or PCA remained significant in the association with END even after adjusting for covariates. This echoes prior results showing an association between brain stem infarct and END [6, 7]. Also, in our patients with END, 15 of 37 patients (40.5%) with PCA lesion and 11 of 19 patients (57.9%) with BA lesion had a brain stem infarct. Moreover, BA lesion in our total cohort was associated with BAD (12 of 47 patients with BA lesion, 25.5% versus 9 of 469 patient without, 1.9%, *P* < 0.001), which had a significant relationship to END presently and in prior studies [7, 18, 19].

The incidence of END in our patients was 19.0%, which was within the range of the reported incidence of 6.8–35% [3, 22]. This wide variation is presumed to be attributable to the difference in the definition of END, onset-visit time of included patients, and the proportion of stroke types in each study.

Similar to prior studies, patients with END had a higher mortality and poor functional recovery [1, 2]. However, there was no difference in the occurrence of further vascular events between patients with and without END, although END was associated with IAS and thus a higher atherosclerotic burden, as shown previously. This might be due to aggressive medical treatment and risk factor management for post-stroke survivors with IAS among our patients. In particular, dual antiplatelet therapy (aspirin and clopidogrel) with high-intensity statin was prescribed in those patients for several months according to a guideline from the American Heart Association and American Stroke Association [23]. This practice is further supported by the results of a recent study showing that a short course (a month) of dual antiplatelet therapy reduced END and stroke recurrence in patients with acute LAA stroke [24]. Noticeably, the effect of dual antiplatelet therapy was higher in patients with posterior circulation stroke and basilar stenosis in that study. Accordingly, dual antiplatelet therapy can be positively considered for patients with symptomatic basilar artery stenosis likely to have END.

Our study has several limitations. First, it was based on a single center data with a small number of patients, potentially leading to selection bias. Thus, our results might not be generalizable to other stroke populations. Second, we used 1.5 T MRA to determine arterial stenosis. Because this modality cannot discern an atherosclerotic plaque or subtle stenotic segment, some cases of BAD may have been misclassified as other stroke types, especially lacune [25]. Lastly, we could not completely distinguish atherosclerotic lesion from cardiac embolus in patients with cardioembolic stroke. In this study, complete occlusion relevant to cardioembolic infarct was excluded in determining distribution of stenosis because higher prevalence of cardiac embolus than atherosclerosis has been reported in such lesions [26, 27]. Even so, the completely occluded lesions might possibly include underlying atherosclerotic lesion. In addition, embolus within a cerebral artery may also cause vessel narrowing if not completely occlusive, which may mimic a stenosis on MRA. Thus, susceptibility-weighted image may be of help (which was not analyzed in this study) to determine a thrombus signal [28].

## Conclusions

Pre-stroke distribution of atherosclerotic stenosis (IAS) as well as stroke subtypes of LAA (or BAD) may determine neurologic worsening during the early stage of ischemic stroke. IAS may limit collateral channels, leading to stroke progression in border zone, and interrupt the recovery of blood flow in the penumbra zone, resulting in lesion growth. More research is needed to confirm these results.

## Supporting information

S1 File Patient data including all and END patient data. (XLS)

S1 Table Logistic regression analysis for poor functional outcome. (DOCX)

S2 Table Comparison of clinical characteristics between patients with and without intracranial atherosclerotic stenosis. (DOCX)
