# Supplementary material for: Distribution of atherosclerotic stenosis determining early neurologic deterioration in acute ischemic stroke
Source: PLoS One. 2017 Sep 25;12(9):e0185314. doi: 10.1371/journal.pone.0185314 (PMC5612689; doi:10.1371/journal.pone.0185314)
Supplement: S1 Table — (DOCX) [file pone.0185314.s002.docx]

S1 Table. Logistic regression analysis for poor functional outcome (6-month mRS≥3)

|  | Univariate |  | Multivariate |  |
| --- | --- | --- | --- | --- |
|  | OR (95% CI) | *P* | OR (95% CI) | *P* |
| Age ≥65 years | 3.385 (2.223-5.154) | <0.001 | 3.098 (1.311-7.319) | 0.010 |
| Female | 2.403 (1.656-3.487) | <0.001 | 1.475 (0.691-3.147) | 0.315 |
| Hypertension | 1.249 (0.844-1.847) | 0.267 |  |  |
| Diabetes | 1.314 (0.890-1.940) | 0.169 |  |  |
| Hyperlipidemia | 1.131 (0.786-1.627) | 0.507 |  |  |
| Previous stroke | 1.840 (1.150-2.943) | 0.011 | 0.744 (0.324-1.707) | 0.486 |
| Ischemic heart disease | 1.161 (0.753-1.792) | 0.499 |  |  |
| Congestive heart failure | 2.509 (1.502-4.193) | <0.001 | 1.632 (0.704-3.783) | 0.254 |
| Valvular heart disease | 1.094 (0.702-1.703) | 0.691 |  |  |
| Atrial fibrillation | 1.530 (1.052-2.226) | 0.026 | 1.186 (0.524-2.683) | 0.682 |
| Current smoking | 0.387 (0.243-0.615) | <0.001 | 0.576 (0.192-1.725) | 0.324 |
| Heavy alcohol consumption | 0.340 (0.189-0.613) | <0.001 | 0.537 (0.110-2.626) | 0.442 |
| Initial NIHSS score | 1.231 (1.184-1.279) | <0.001 | 1.235 (1.170-1.304) | <0.001 |
| Intracranial atherosclerotic stenosis | 3.072 (2.109-4.475) | <0.001 | 2.495 (1.116-5.575) | 0.026 |
| Stroke subtype (cardioembolism) | 1.013 (0.698-1.470) | 0.946 |  |  |
| Early neurologic deterioration | 8.704 (5.253-14.425) | <0.001 | 3.383 (1.281-8.937) | 0.014 |

OR, odds ratio; CI, confidence interval; NIHSS, National Institutes of Health Stroke Scale; mRS, modified Rankin scale
