# Supplementary material for: Distribution of atherosclerotic stenosis determining early neurologic deterioration in acute ischemic stroke
Source: PLoS One. 2017 Sep 25;12(9):e0185314. doi: 10.1371/journal.pone.0185314 (PMC5612689; doi:10.1371/journal.pone.0185314)
Supplement: S2 Table — (DOCX) [file pone.0185314.s003.docx]

S2 Table. Comparison of clinical characteristics between patients with and without intracranial atherosclerotic stenosis (IAS): n (%)

|  | IAS (+)  N=221 | IAS (-)  N=295 | *P* |
| --- | --- | --- | --- |
| Age ≥65 years | 160 (72.4) | 160 (54.2) | <0.001 |
| Women | 117 (52.9) | 129 (43.7) | 0.038 |
| Hypertension | 175 (79.2) | 171 (58.0) | <0.001 |
| Diabetes | 97 (43.9) | 60 (20.3) | <0.001 |
| Hyperlipidemia | 130 (58.8) | 135 (45.8) | 0.003 |
| Current smoking | 59 (26.7) | 79 (26.8) | 0.983 |
| Previous stroke | 42 (19.0) | 44 (14.9) | 0.217 |
| Ischemic heart disease | 65 (29.4) | 48 (16.3) | <0.001 |
| Atrial fibrillation | 45 (20.4) | 139 (47.1) | <0.001 |
| Valvular heat disease | 30 (13.6) | 78 (26.4) | <0.001 |
| Heavy alcohol consumption | 30 (13.6) | 57 (19.3) | 0.084 |
| Congestive heart failure | 33 (14.9) | 36 (12.2) | 0.367 |
| Metabolic syndrome | 142 (64.3) | 101 (34.2) | <0.001 |
| Stroke classification |  |  | <0.001 |
| Large artery atherosclerosis | 105 (47.5) | 11 (3.7) |  |
| Cardioembolism | 14 (6.3) | 66 (22.4) |  |
| Lacune | 30 (13.6) | 169 (57.3) |  |
| Two or more | 63 (28.5) | 19 (6.4) |  |
| Cryptogenic | 8 (3.6) | 26 (8.8) |  |
| Other causes | 1 (0.5) | 4 (1.4) |  |
| Initial NIHSS score | 7.6±7.9 | 6.3±8.2 | 0.064 |
| Poor functional outcome | 108 (48.9) | 70 (23.7) | <0.001 |

Poor functional outcome indicates scores ≥3 on 6-month modified Rankin scale.

P was calculated by Chi-square test or independent t-test.
